# Remote Data Retrieval for Bioinformatics Applications: An Agent Migration Approach

**DOI:** 10.1371/journal.pone.0020949

**Published:** 2011-06-20

**Authors:** Lei Gao, Hua Dai, Tong-Liang Zhang, Kuo-Chen Chou

**Affiliations:** 1 College of Information Sciences and Technology, Donghua University, Shanghai, China; 2 Shanghai Research and Development Center, Tellabs, Shanghai, China; 3 Research Institute of Highway, Ministry of Transport of China, Beijing, China; 4 Gordon Life Science Institute, San Diego, California, United States of America; University of Vermont, United States of America

## Abstract

Some of the approaches have been developed to retrieve data automatically from one or multiple remote biological data sources. However, most of them require researchers to remain online and wait for returned results. The latter not only requires highly available network connection, but also may cause the network overload. Moreover, so far none of the existing approaches has been designed to address the following problems when retrieving the remote data in a mobile network environment: (1) the resources of mobile devices are limited; (2) network connection is relatively of low quality; and (3) mobile users are not always online. To address the aforementioned problems, we integrate an agent migration approach with a multi-agent system to overcome the high latency or limited bandwidth problem by moving their computations to the required resources or services. More importantly, the approach is fit for the mobile computing environments. Presented in this paper are also the system architecture, the migration strategy, as well as the security authentication of agent migration. As a demonstration, the remote data retrieval from GenBank was used to illustrate the feasibility of the proposed approach.

## Introduction

Due to the exponential growth of biological sequences generated in the postgenomic age [1], a large amount of biology related data is generated and publicly available as web resources [2]. Typically, these resources are accessible only through web-based query interfaces. Analytical methods are often developed on the integration of information from multiple sources in bioinformatics research. This might result in an individual researcher has to do a query at each data source. What's more, these data sources are very possible to have different entries, data formats, query options, and complex results [3].

Fortunately, some approaches have been developed to retrieve data automatically from one or multiple data sources. Buttler and Critchlow propose a meta-data description language to automatically generate wrappers that can extract the appropriate data from each source [3]. Cadag and Tarczy-Hornoch address diverse data retrieval via a simple framework for representing coverage and evidence that operates in parallel with an arbitrary schema, and a language upon which queries on the schema and framework may be executed [4]. Gouy and Delmotte proposes a biological sequence database system named ACNUC, which is able to provide powerful and fast query and extraction capabilities to a variety of nucleotide and protein sequence databases [5]. Lacroix presents a tool to query integrated web data sources composed of a retrieval component based on an intermediate object view mechanism and search views, and an XML engine [6]. Some efforts are done on the integration of biological data available on the web and maintained in diverse sources [7], [8], [9], [10], [11].

However, the most of the above approaches for remote data retrieval require researchers to remain online and wait for returned results. The retrieval mode with the feature of “static service and floating data” not only requires high availability of network connection, but also might result in network overload. Moreover, the existing approaches are not designed to address the remote data retrieval in a mobile network environment, which is welcome by more and more researchers, with the rapid development speed of mobile networks. Researchers often wish that they can obtain retrieval results by their mobile devices so that they are able to promptly make decisions in terms of required information.

To tackle these problems, we integrate the migration mechanism of mobile agents to a multi-agent system to reduce the workload of network transmission, improve the capability of parallel processing, as well as facilitate the flexibility and scalability of the system.

Mobile agents, evolved from autonomous agents [12], [13], [14], have the feature of autonomy, social ability, learning, and most importantly, mobility. They can automatically suspend their executions on one host and migrate to another to resume their computations without tedious and slow network communication [15], [16]. In a mobile network environment, agent migration has a more significant role: due to the resource limitation of mobile devices, agents are sent to some nodes/devices with rich resources for doing some tasks which have high requirements of resources, and then bring results back.

Agent migration has been regarded as a promising approach used in diverse fields of network applications to reduce network load, shorten communication delay, package transmission protocol, and increase flexibility [16], [17], [18]. Recently, a few applications developed using agent migration approach have been seen in a couple of areas, such as wireless ad hot network routing [19], electronic marketplace [20], electronic tour guide [21], distributed information retrieval [22], supply chain management [23], and autonomous software deployment [24]. Some studies have further combined agent migration with ecosystem-inspired evolutionary approaches, aiming to build highly available, ubiquitous, self-managing, and adaptable applications [16], [25], [26]. In general, agent migration approach can be applied to the areas of distributed information retrieval, networks services, electronic commerce, personal assistance, secure brokering, supply chain management, monitoring and notification, information dissemination, and parallel processing [20].

This paper further develops an asynchronous migration mode, where the migration request is initially inserted into a processing queue, rather than immediately addressed. Using this mode, agents can perform their tasks even if the users are mostly in a low-quality connection or disconnection status. Although agent migration approach has many distinct properties that make it a promising direction for data retrieval, it also brings significant security threats, which have become the bottleneck of the development and maintenance of the mobile agent systems [15]. So in the proposed agent migration service, we design a security authentication of agent migration.

The rest of the paper is organized as follows. We first present the architecture of the proposed multi-agent system and the agent migration service. Then, as a demonstration, an application for remote data retrieval in GenBank [27] is developed to illustrate the feasibility of the proposed approach. Finally, this paper concludes our research efforts.

## Methods

### 1. System Architecture

**Figure 1:**
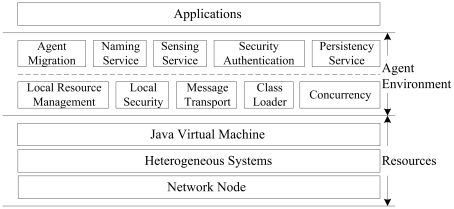
The architecture of the proposed multi-agent system.

The proposed multi-agent system is fully implemented using Java. This architecture (as shown in Figure 1) is regarded as a three-layer model, including: (1) resources layer, (2) agent environment layer, and (3) applications layer.

Each system is hosted in a network node (e.g., a mobile phone or a supercomputer) and runs upon a Java virtual machine (JVM), which is built on an active operating system in the node. So the resources layer consists of all kinds of basic resources available in the network node. The applications layer establishes a friendly interface between users and the proposed system. Many services in this layer are provided through web pages and can be implemented by JavaBean, JSP, Servlet, and so on.

In the middle of the above two layers, the agent environment is the runtime environment for deploying and executing agents. Upon the resource layer, the agent environment layer provides a bunch of low-level functional modules which can help agents fully use the resources. The low-level functional modules include local resource management, local security, message transport, class loader, and concurrency. Then upon the low-level functional modules, the agent environment layer provides a set of general-purpose runtime services that are frequently used by agents, including naming service, sensing service, security authentication service, persistency service, and agent migration service. These services alleviate agents from low-level operations and allow agents to be lightweight by separating them from some routine work. This layer of the proposed multi-agent system is implemented based on the ENGM platform. The discussion on design philosophy, layer analysis, service design, functional merits and message-based communication of the ENGM system is beyond the scope of this paper. Readers are referred to Refs. [13], [28], [29].

### 2. Agent Migration Service

Agent migration can help agents: (1) approach data sources or service requests, and reduce network communication workload; (2) utilize rich resources in destination nodes; (3) encapsulate a suitable transmission protocol for establishing connection in accordance to the applied protocol; and (4) find useful partner agents in other network nodes, build new relationships, and improve service quality. The proposed multi-agent system has two migration modes: synchronous and asynchronous migration. In the synchronous mode, the migration request of a mobile agent is processed immediately. However, if the agent migration service of the system finds the destination node is unreachable, it will send an error message to its administrator and let him/her determine its next step. In the asynchronous migration mode, the migration request is initially inserted into a processing queue and not immediately processed. The asynchronous migration mode is required in some tough situations, such as when the destination node is unreachable, when executing a task without persistent connection, or when a mobile agent in a node with persistent connection is waiting for returning to an offline node.

The agent migration service is responsible for sending/receiving an agent to/from another host. It serializes both agent code and execution state into streams/bit-blobs which are suitable for network transfer and persistent storage. The persistency service is responsible for storing serialized agents into persistent storage. It minimizes resource consumption while agents wait for executing a task. In addition, the persistency service offers fault tolerance by duplicating and storing agent copies before starting critical operations.

Two key technologies: migration strategy and security authentication of agent migration are presented as follows.

#### 2.1. Migration Strategy

**Figure 2:**
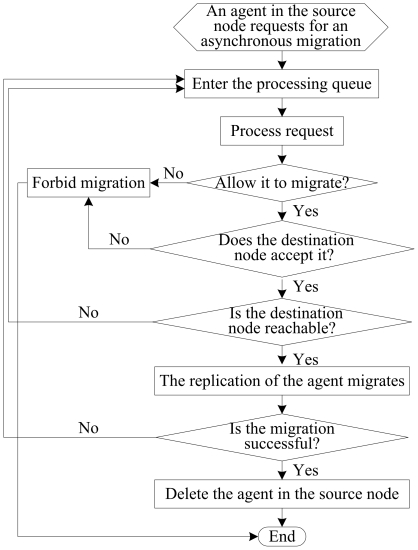
The flowchart of an agent asynchronous migration.

In this section, as a demonstration of agent migration strategy, a flowchart of agent asynchronous migration is shown in Figure 2. When a migration request is under processing, the agent migration service of the multi-agent system checks migration conditions in the source node, deciding to allow the agent to migrate or not. In addition, the agent migration service judges whether the destination node agrees the migration, the destination node is reachable, and the migration is successful, then take different actions. Once the migration is successful, the replication of the agent in the destination node will execute its task and the original agent will be deleted in the source node.

#### 2.2. Security Authentication of Agent Migration

The agent migration approach also brings significant security concerns, among which the primarily important problems are the ones between a mobile agent and its platforms. Existing mobile agent systems mostly utilize user information stored in external directory services to authenticate an external access. However, the resource limitation of mobile devices restricts the communication between external directory services and themselves. So a security authentication of agent migration must be done locally and not involve an external directory service.

**Figure 3:**
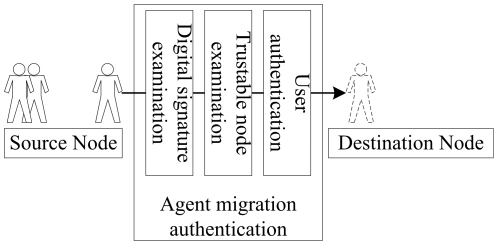
A demonstration of agent migration authentication.

We design a security authentication of agent migration based on java virtual machine (JVM). Figure 3 demonstrates the designed security authentication of agent migration, which includes digital signature exanimation, trustable node examination, and user authentication. To the agent that migrates to a new node successfully, the local security module in the node will authenticate and monitor its access to local resources.

The digital signature exanimation is used to confirm if a migrating agent (i.e., migration codes) changes. An application developer creates and signs message abstract where a signature is encrypted digital data. The JVM decrypts the message abstract of the migration codes and compares it with the abstract decrypted using a public cryptographic key. If two abstracts match, there is no change in the migrating agent. In this examination, the public cryptographic key should be registered locally before receiving remote migration codes.

Note that even if the migration codes are trustable, the remote program may be harmful. The trustable node examination is based on JVM, which creates protection domain for executing program and restricts the privilege of the remote program. When an agent migrates to a new node, the Java class loader of the JVM in the node will examine the creator of the agent. If the creator is not in the local list (a JVM maintains a list of trustable nodes), the JVM will assign the least privilege to execute the migration codes.

The security authentication service also maintains a user authentication table, which is used to authenticate mobile agents that migrate from other hosts. An access privilege is created based on the agent metadata, the authentication result of the source node, and local security requirements. Once the system receives the migration request from an unknown node or an unmarked agent, the security authentication will send a message to its administrator (terminal user) and let the administrator determine how to deal with the situation. According to the decision made by the administrator, the security authentication offers agents different access privileges.

## Results and Discussion

### A Case Study of Remote Data Retrieval Using Agent Migration

We apply the migration service described above to remote data retrieval in GenBank, which is the NIH genetic sequence database, an annotated collection of all publicly available DNA sequences. In this case, we want to retrieve all genome sequences of spike (S) protein of severe acute respiratory syndrome coronavirus (SARS-CoV) [30] from the GenBank database. The idea is according to the record format in Genbank is relatively invariable, sending migration programs to the website and capturing effective information locally from the web tags.

**Figure 4:**
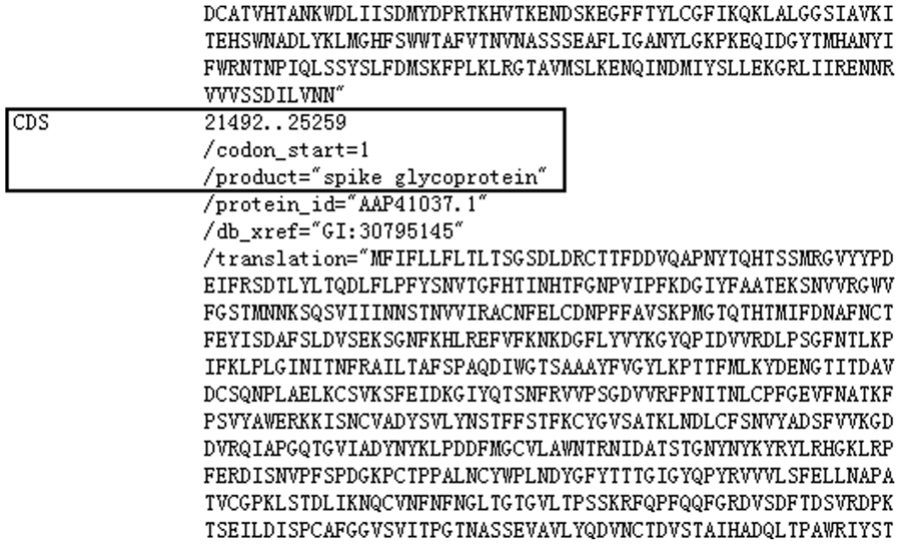
A demonstration of the start and end positions of a genome sequence of S protein.

After the mobile agent is allowed to execute tasks in the destination node (a GenBank server), firstly, the mobile agent is required to obtain the start and end positions of a genome sequence of S protein by finding the information “/product = ‘spike glycoprotein’”, as shown in the blank box of Figure 4.

**Figure 5:**
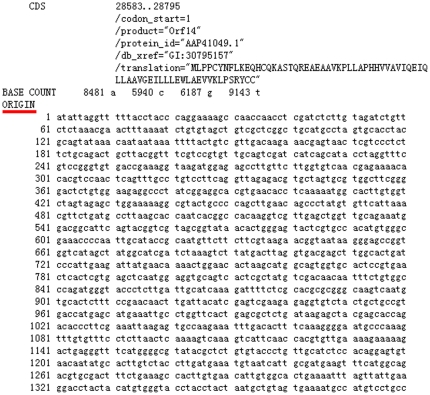
The start of a genome sequence under “ORIGIN” tag.

Secondly, the agent needs to capture sequence data under “ORIGIN” tag, which stands for the start of a sequence, as shown in Figure 5.

**Figure 6:**
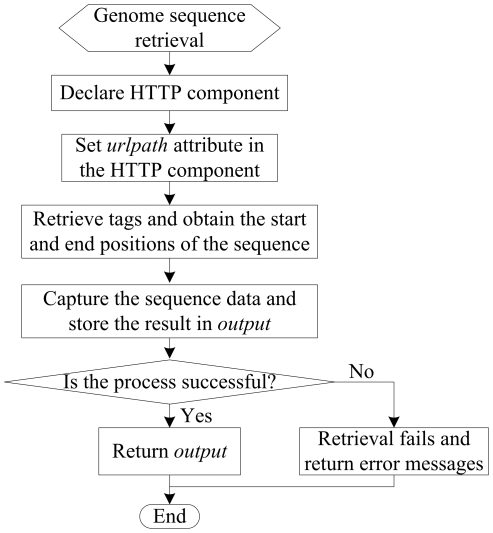
The flowchart of data retrieval of the mobile agent.

The flowchart of data retrieval of the mobile agent is shown in Figure 6. The agent is required to declare a HTTP component to locate the data source, then capture and store the information. Next, the agent will check the information obtained, if the process is verified as a successful case, the information captured will be returned to client terminals; otherwise, error messages will be sent to the terminals.

In the implementation of the case study, when a mobile agent is required to migrate, it invokes *requestMigration*() method to request an asynchronous migration. Then the multi-agent system processes the request by executing *preDeliverAgent*() method, and build a connection with the destination host by remotely invoking *preReceiveAgent*() method in that host through RMI over Internet inter-orb protocol (RMI-IIOP) [31]. Furthermore, the multi-agent system sends a request for transferring an agent to the system in the destination host. After obtaining a positive response from the destination host, the system in the source host starts to transfer the replication of the agent. If the transfer is successful, a message will be sent to close the connection, register the information to the destination host, make the agent reloaded in the destination host, and inform the source host to delete the agent; otherwise, the request will enter the processing queue in the source host. Parts of *preDeliverAgent*() method and *preReceiveAgent*() method are shown as follows,

public void preDeliverAgent (Agent agent, String desAddress) {

String agentId = agent.getAgentID(); // obtain the ID of the agent;

String address = desAddress; // the address of the destination host;

String rmiiiopAddress = “//”+address+“/preReceiveAgent”;

Receiver receiver = (Receiver) Naming.lookup (rmiiiopAdddress); // look for *preReceiveAgent*() method;

boolean migrate  =  receiver.preReceiveAgent (host, port, agentId); // invoke *preReceiveAgent*();

…

}

public boolean preReceiveAgent (String host, int port, String agentId) {

Connection con = new Connection (host, port); // build a connection;

ObjectOutputStream out = new ObjectOutputStream(con.getOutputStream()); // create output stream;

ObjectInputStream input = new ObjectInputStream(con.getInputStream()); // create input stream;

out.writeObject(agentId); // send agent ID to the host;

String agentName = (String) input.readObject(); // obtain agent name;

// communicate with the source host to examine whether the agent is ready to migrate

…

// receive the agent if it is ready, otherwise return error messages

…

con.close(); // close connection;

this.server.registerAgent(agentName, agentId); // register the agent;

this.server.startAgent(agentId); // reload the agent;

}

**Figure 7:**
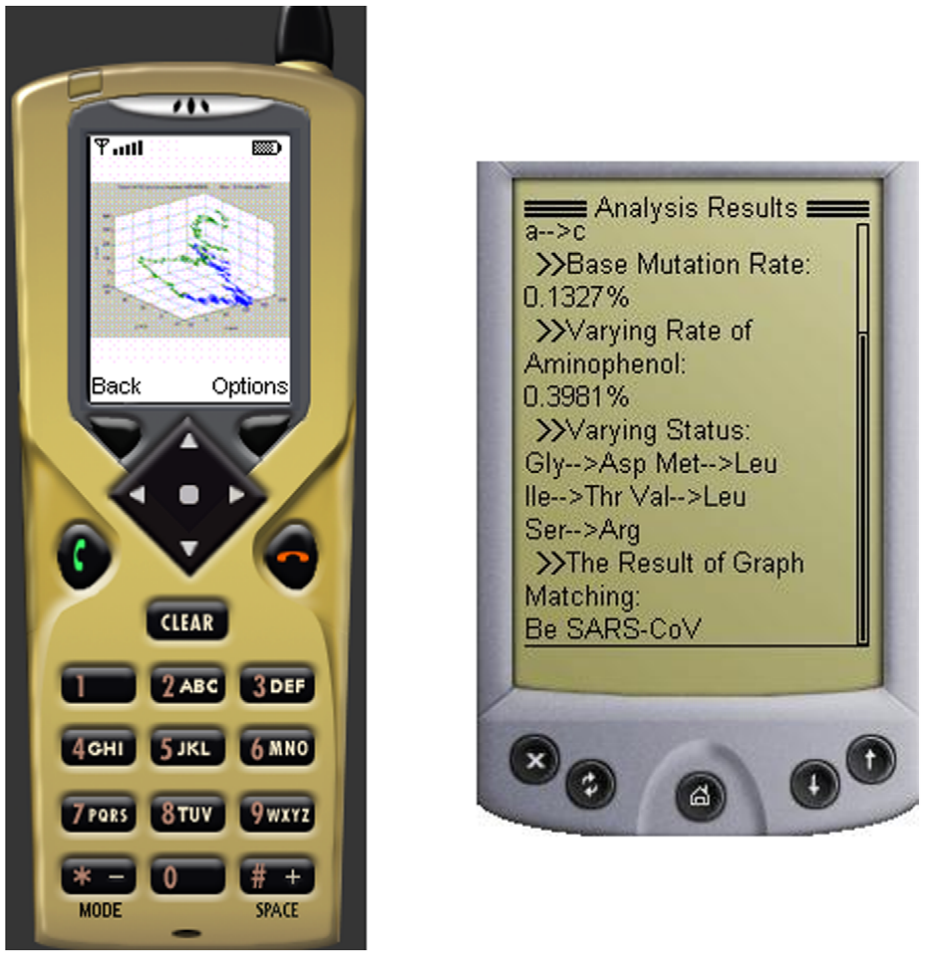
The returned results in mobile device emulators.

Furthermore, we can utilize rich resources in destination nodes or third-party platforms to deal with obtained data and do some analyses, then return the processed results to mobile devices with limited resources. For example, in the above case, the genome sequences of S protein can be further analyzed using the approach proposed in Ref. [30] and the agent only needs to return the analysis results. Figure 7 demonstrates the returned analysis results in two mobile device emulators.

This paper proposes an agent migration approach to fill in the gap that existing approaches have not addressed: retrieving remote data in a low-quality network environment, especially unstable mobile computing environments. The ability of migration offers mobile agents a means to overcome the high latency or limited bandwidth problem by moving their computations to required resources or services. The proposed approach can also overcome the resource limitation of mobile terminals and release mobile users from keeping online persistently. The system architecture and the migration service are given in details. A remote data retrieval in GenBank was used to illustrate the feasibility of the proposed approach.

The agent migration approach can also be applied to retrieving non-data web resources, for example, sending mobile agents to some bioinformatics web servers (e.g., [32], [33], [34], [35], [36], [37]) and retrieving analysis results to mobile devices. Since user-friendly and publicly accessible web-servers represent the future direction for developing practically more useful models, simulated methods, or predictors [34], [38], we shall make efforts in our future work to provide a web-server for the approach presented in this paper.
